# Hydrogel Biomaterials for Application in Ocular Drug Delivery

**DOI:** 10.3389/fbioe.2020.00228

**Published:** 2020-03-20

**Authors:** Courtney R. Lynch, Pierre P. D. Kondiah, Yahya E. Choonara, Lisa C. du Toit, Naseer Ally, Viness Pillay

**Affiliations:** ^1^Wits Advanced Drug Delivery Platform Research Unit, Department of Pharmacy and Pharmacology, School of Therapeutics Sciences, Faculty of Health Sciences, University of the Witwatersrand, Johannesburg, South Africa; ^2^Division of Ophthalmology, Department of Neurosciences, School of Clinical Medicine, Faculty of Health Sciences, University of the Witwatersrand, Johannesburg, South Africa

**Keywords:** biopolymers, ocular drug delivery, hydrogel, nanotechnology, biomaterials, safety by design

## Abstract

There are many challenges involved in ocular drug delivery. These are a result of the many tissue barriers and defense mechanisms that are present with the eye; such as the cornea, conjunctiva, the blinking reflex, and nasolacrimal drainage system. This leads to many of the conventional ophthalmic preparations, such as eye drops, having low bioavailability profiles, rapid removal from the administration site, and thus ineffective delivery of drugs. Hydrogels have been investigated as a delivery system which is able to overcome some of these challenges. These have been formulated as standalone systems or with the incorporation of other technologies such as nanoparticles. Hydrogels are able to be formulated in such a way that they are able to change from a liquid to gel as a response to a stimulus; known as “smart” or stimuli-responsive biotechnology platforms. Various different stimuli-responsive hydrogel systems are discussed in this article. Hydrogel drug delivery systems are able to be formulated from both synthetic and natural polymers, known as biopolymers. This review focuses on the formulations which incorporate biopolymers. These polymers have a number of benefits such as the fact that they are biodegradable, biocompatible, and non-cytotoxic. The biocompatibility of the polymers is essential for ocular drug delivery systems because the eye is an extremely sensitive organ which is known as an immune privileged site.

## Introduction

There have been many recent advancements made in the delivery of drugs to the eye, a site that is challenging to treat. The eye is a relatively isolated organ within the body, with many barriers and mechanisms that limit the entry of foreign substances into the eye. These include, among others, the cornea, blinking reflex, blood-aqueous barrier, blood–retina barrier, and the nasolacrimal drainage system. Collectively, these systems make the delivery of drugs to both the anterior and posterior segment of the eye more difficult (Patel et al., 2013). Novel drug delivery systems are constantly being developed to overcome the low bioavailability observed in many conventional ophthalmic formulations; these novel systems include the development of hydrogels.

Hydrogels have been largely investigated within the medical industry for a number of purposes; including drug delivery and tissue engineering. These systems are composed of cross-linked polymers which are capable of swelling when placed in water or an aqueous environment. Hydrogels have been researched in terms of drug delivery because they are able to hold, within the cross-linked matrix, a number of different substances. These range from hydrophobic and hydrophilic molecules to both micro- and macromolecules (Kang Derwent and Mieler, 2008). An example of the effectiveness of hydrogels in drug delivery is shown in the article by Li et al. (2018) where the delivery of antibiotics by hydrogel systems was discussed. It was highlighted how hydrogels are able to deliver antibiotics to a local site (overcoming the severity of side effects often seen with systemic administration), offer controlled release of the active ingredient, and have better biocompatibility than conventional drug delivery systems (Li et al., 2018). These benefits can be translated into the development of hydrogel systems for the delivery of drugs to the eye.

**FIGURE 1:**
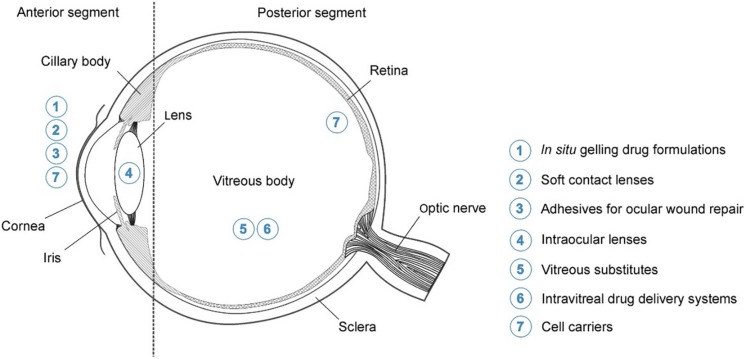
Highlighting the potential application for hydrogels in ocular drug delivery. These include the delivery of drugs to both the anterior and posterior segments of the eye which will aid in overcoming the physiological barriers. Possible topical formulations for delivery to the anterior segment include systems which gel upon application (*in situ* gelling formulations) and contact lenses. Posterior segment formulations include intravitreal injections, which are made more effective by hydrogel technology, and cell carrier systems (adapted with permission from Kirchhof et al., 2015).

Due to the fact that hydrogels are so versatile and are able to be modified to exploit the environment and function they are being designed for; these systems are highly advantageous in the effective delivery of drugs to the eye (Kang Derwent and Mieler, 2008). Figure 1 indicates the various potential applications for hydrogels in ocular drug delivery.

Hydrogels have been shown to alter the drug release profiles of a formulation (to a sustained drug release profile), largely due to the swelling rate and water adsorption properties of the biotechnology platform. This swelling rate of the hydrogel can be induced as a response to a change in the environment into which the hydrogel is placed; these are known as “smart” or stimuli-responsive hydrogels. The stimulus can be chemical or physical and allows for the development of drug delivery systems which are regulated by the body. In addition, these “smart” hydrogels are able to respond to external stimuli such as in the process of iontophoresis (Fathi et al., 2015).

Through the development of stimuli-responsive hydrogel systems, not only are researchers able to overcome the issues of low bioavailability and rapid removal from administration site which is currently seen with conventional formulations, they are also able to do so without comprising on patient comfort. These delivery systems are able to be administered as a liquid and then form a gel once in contact with the eye (Hamcerencu et al., 2020). This is an important factor to consider in terms of patient compliance as patients are less likely to make use of an ophthalmic formulation if it is difficult to administer which is often the case with formulations that are highly viscous such as ointments (Singh et al., 2019).

Polymers have received much attention for use in drug delivery, and more specifically ocular drug delivery, over recent years. Although there are countless polymers available, this review article focuses on those which occur naturally, also known as biopolymers. These specific polymers offer the beneficial properties of being biodegradable, biocompatible, and non-cytotoxic. They also have the advantages of being readily available, renewable, and less expensive in comparison to synthetic polymers (Oh et al., 2009).

## Physiological Ocular Barriers and Defense Systems Which Impact Drug Delivery

There are many challenges when it comes to effective delivery of drugs to the eye. Many of these are as a result of the barriers and mechanisms present within the eye which are designed to protect it from foreign particles and substances. A brief overview of the major ocular defense mechanisms is discussed below.

The first defense mechanism found in the eye is pre-corneal factors which result in the low bioavailability of topically applied ocular formulations. These include the blinking reflex, high tear turnover rate, and the lacrimal drainage of the solution. The cul-de-sac of the eye can hold approximately 30 μl of an administered eye drop. However, majority of this is removed within 15–30 s after the drops have been administered (Gaudana et al., 2010). Considering these factors, drug delivery systems need to be developed that are able to improve the retention of the formulation at the administration site. Consequently, this will improve the penetration of the active ingredient into the eye. Both hydrogel systems and mucoadhesive biopolymers could furnish formulations with these much-needed advantages (Biro and Aigner, 2019).

One of the major barriers to foreign substance entry into the eye is the multiple layers through which substances must pass through in order to penetrate into the target tissues. These layers include the cornea and the conjunctiva, among others. The cornea is located in the anterior segment of the eye and it made up of six layers: the epithelium, Bowman’s membrane, stroma, Dua’s layer, Descemet’s membrane, and the endothelium (Ludwig, 2005; Dua et al., 2013). It is one of the main penetration-limiting layers in terms of drug delivery. This layer is highly lipophilic which largely prevents the entry of hydrophilic molecules into the eye (Moiseev et al., 2019).

The conjunctiva is a highly vascularized membrane that covers most of the anterior aspect of the eye. This high vascularity means that, although it can be used for the delivery of hydrophilic and large molecules, a large portion of the administered drug will be removed via the conjunctiva and enter systemic circulation before penetrating into the eye. This is also one of the main reasons why topically administered drugs are not able to reach the posterior segment of the eye in effective concentrations (Willoughby et al., 2010).

**FIGURE 2:**
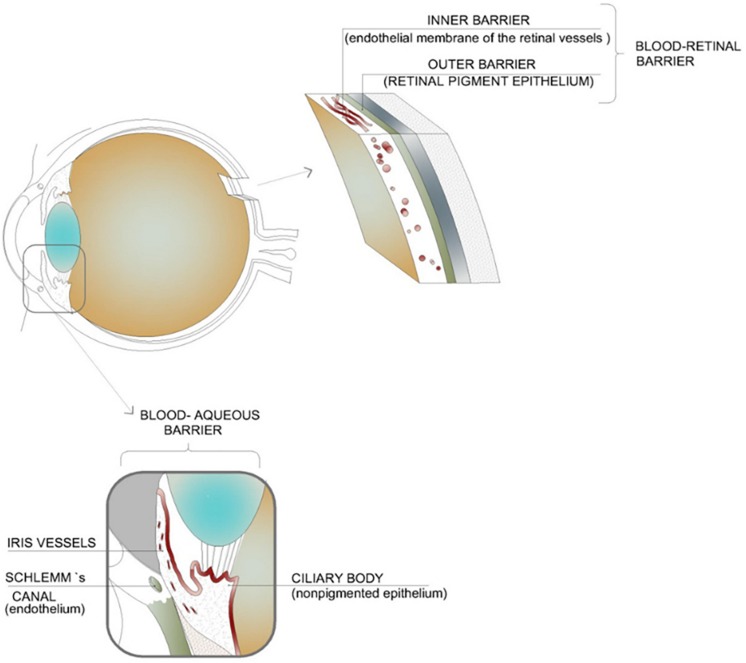
Illustration of the blood–ocular barriers which inhibit the movement of active ingredients into the eye from systemic circulation; namely, the blood–aqueous barrier and the blood–retinal barrier. These barriers result in the need for high systemic dosages of drugs in order to achieve an adequate concentration within the intended tissues. This high dosage can lead to unwanted side effects (adapted with permission from Occhiutto et al., 2012).

The eye is composed of two segments; the anterior segment (composed of the aqueous humor, conjunctiva, cornea, iris, ciliary body, and lens) and the posterior segment (composed of the choroid, optic nerve, retina, sclera, choroid, and vitreous humor). Each segment is susceptible to a range of conditions and each poses its own challenges when it comes to drug delivery (Souto et al., 2019). There are two blood-ocular barriers; the blood-aqueous barrier and the blood-retinal barrier. These largely prevent the entry of substances into the eye from systemic circulation. Although systemic administration has been considered as a route for drugs needed in the posterior segment of the eye, the dose needed is often high which leads to unwanted side effects (Nettey et al., 2016). Figure 2 highlights the blood-ocular barriers in addition to the tissues which comprise these barriers.

When a formulation is applied to the surface of the eye (i.e., topical administration), it is rapidly removed through the blinking reflex and nasolacrimal drainage. This drainage system removes the drug from the eye via the nasolacrimal duct. It then enters the nose and is absorbed by the nasal mucosa where it enters into systemic circulation. This is another factor which furthers the low bioavailability of topical applied ophthalmic preparations (Rajasekaran et al., 2010).

Hydrogels have been shown to increase the residence time of an active ingredient, allowing more time for it to diffuse through the layers of the eye. This plays a major role by increasing the bioavailability of topically administered ophthalmic formulations (Vashist et al., 2014). Due to the increased viscosity of a hydrogel system, it is also better able to withstand the clearance of the formulation due to blinking, further improving the bioavailability (Li Z. et al., 2013).

Biopolymers have also been shown to help overcome these barriers to drug delivery. Some, such as chitosan, have inherent mucoadhesive properties which allow the formulation to remain at the administered site for a longer period of time (Fulgencio et al., 2012). Cellulose derivatives have also been used to enhance the viscosity of a formulation, thereby preventing it from being washed away from the ocular surface too rapidly (Rajasekaran et al., 2010).

## Current Commercial Formulations Utilized for the Delivery of Drugs to the Eye

There are many formulations currently on the market which are designed to treat ophthalmic conditions. These range from anterior segment conditions such glaucoma, bacterial conjunctivitis, and post-operative inflammation to posterior segment conditions such as neovascular age-related macular degeneration, uveitis, and macular edema (Sultana et al., 2006b; Bao et al., 2017; Kaji et al., 2018). Each of the drug delivery systems discussed below has distinctive disadvantages when it comes to the effective delivery of drugs to the eye. It has been shown that the inclusion of hydrogels into the drug delivery system has been able to overcome some of these challenges, as is highlighted by the various studies included below.

Currently, the most common dosage form used to treat ocular conditions is eye drops. These formulations can be solutions or suspensions. However, although they are the first line treatment, there are many limitations to their use. These range from low bioavailability and rapid clearance from the administration site, to poor patient compliance (Yellepeddi and Palakurthi, 2016). Active ingredients in eye drops are not able to penetrate through to the posterior segment of the eye and thus are mainly used to treat anterior segment conditions (Urtti, 2006).

Conventional, commercially available eye drops often have frequent dosing schedules (ranging from daily to multiple times a day) and, in the case of chronic conditions such as glaucoma, require the patient to use them on a long-term basis. This can lead to unwanted side effects, which, for example, has been seen with latanoprost eye drops (daily administered dose of one drop). These side effects can cause patients to stop using their medications as prescribed, or to not use them at all. This is another reason why novel drug delivery systems such as hydrogels are needed; to reduce the frequency of dosing, reduce side effects and be patient-friendly enough so that patients will use them for an extended period of time if need be (Cheng et al., 2016).

In a recent article written by Yadav et al. (2019), it was highlighted how pre-corneal factors lead to the low absorption of ocular active ingredients used to treat glaucoma, administered as eye drops. These factors, such as tear turnover rate and the drainage of the formulation from the administration site, result in a 70–80% loss of the amount of drug which is administered. It was also highlighted how the frequent dosing schedules of eye drops can cause damage of to the eye. The consideration of ointments has been made, as these formulations have a higher viscosity and are not as rapidly drained from the eye as a liquid formulation. However, ointments are known to cause blurred vision when administered which leads to poor patient compliance (Yadav et al., 2019).

Posterior segment conditions are generally treated using sub-tenon, intravitreal, or systemic administration. However, each of these routes also comes with challenges of its own. One of the main objectives in the development of new drug delivery systems for the posterior segment is to reduce the invasiveness of the formulations which are currently used. For example, anti-vascular endothelial growth factors (anti-VEGFs) are used to treat a number of posterior segment conditions, namely those affecting the retina such as myopic choroidal neovascularization and diabetic macular edema. However, anti-VEGF is currently only able to be administered via intravitreal injections as the molecules are large and hydrophilic which prevent them from penetrating through the various barriers. This highlights the need for new technologies and drug delivery systems which are able to deliver molecules such as anti-VEGF without frequent, invasive injections (Wong and Wong, 2019).

Intravitreal injections are able to deliver a high concentration of the drug directly into the vitreous of the eye but are invasive and pose risks such as retinal detachment, vitreous hemorrhage, and endophthalmitis. The chances of these happening increase with the frequency of administration (Urtti, 2006; Gaudana et al., 2009). The use of hydrogels as intravitreal injections, with their extended drug release profiles, can delay the frequency of intravitreal injections, thus lowering the chances of the aforementioned risks occurring. Table 1 highlights the formulations which are currently used to treat ophthalmic conditions, both in the anterior and posterior segment of the eye. A brief breakdown of the disadvantages of each of the formulations is also given.

**TABLE 1:** Current ophthalmic formulations which are used to treat anterior and posterior segment conditions.

**Administration**	**Preparations**	**Conditions**	**Disadvantages**	**References**
Topical preparations	Eye drops (solutions and suspensions)	Glaucoma, dry eye, infectious keratitis, conjunctivitis anterior uveitis, post-operative inflammation.	Low bioavailability, frequent dosing regimen, preservatives often used in formulation.	Sultana et al., 2006b; Gupta et al., 2013
	Ointments and gels	Open-angle glaucoma, dry eye, blepharitis bacterial conjunctivitis.	Poor content uniformity, Known to cause blurred vision when applied, inaccurate dosing, eyelid matting.	Li J. et al., 2013; Bao et al., 2017; Shen et al., 2018
	Contact lenses	Post-operative barrier for protection of cornea, pain relief, protection of cornea following injury.	Lack of controlled release mechanism, drug is released from the system very quickly.	Lim et al., 2001; Tieppo et al., 2012
Intraocular preparations	Intravitreal injections	Neovascular age-related macular degeneration, diabetic macular edema, proliferative diabetic retinopathy choroidal neovascularization.	Invasive procedure for the patient, possible complications (retinal detachment, endophthalmitis, subconjunctival hemorrhage, and cataract formation)	Kaji et al., 2018
	Subtenon injections	Macular edema, intermediate uveitis.	Active ingredient must cross multiple barriers before reaching the retina, occasionally less effective than intravitreal injections	Bonfioli et al., 2005; Ozdek et al., 2006; Thomas et al., 2006
	Intraocular implants	Uveitis, cytomegalovirus retinitis, diabetic macular edema.	Invasive surgical insertion and removal (if the implant is not biodegradable), predetermined drug release rates	Wang et al., 2013; Yasin, 2014

*These formulations, both topical and intraocular, each have a number of disadvantages or challenges in terms of drug delivery which can be overcome by hydrogel systems.*

## Characterization Between Physically and Chemically Cross-Linked Biotechnology Hydrogel Systems

As previously mentioned, hydrogels are formed from polymers through a process known as cross-linking. Cross-linking occurs when one polymer chain is linked to another chain via a bond, either through a chemical or physical process. It is these bonds which give hydrogels their stability and multidimensional network structure. The process of cross-linking a hydrogel can have an impact on its physical properties such as elasticity, viscosity, and solubility (Maitra and Shulka, 2014).

Although chemical and physical cross-linking methods each have their own advantages and disadvantages, it is worth noting that physically cross-linked hydrogels do not employ agents containing reactive functional groups which may cause inflammatory responses *in vivo.* However, these hydrogels also result in limited control over how the hydrogel is degraded within the body and, if the physical bonds are not strong enough, the inevitable dilution within the body can negatively impact the mechanical integrity of the hydrogel (Patenaude et al., 2014).

### Hydrogels Which Are Cross-Linked Through Physical Bonds

Physical bonding occurs through interactions between the polymer chains such as ionic bonding, Van der Waals forces, hydrogen bonding, or hydrophobic forces. Due to these types of bonds, the hydrogels formed through physical bonds are known to be reversible and have a degree of instability (Trombino et al., 2019). The hydrogels formed through physical interactions are generally less stable than those formed through chemical interaction as these bonds are susceptible to formation and breakage when there are changes in pH, temperature, and ionic strength. However, this can be a favorable characteristic if the desired outcome is a reversible hydrogel (Kirchhof et al., 2015).

### Hydrogels Which Are Cross-Linked Through Chemical Bonds

Chemically formed hydrogels are known as “permanent” hydrogels due to the covalent bonds which form between polymer chains. These systems allow more stability and maintain their structure better than the physical hydrogels (Trombino et al., 2019). However, it is important that the cross-linking agent can be removed completely from the hydrogel or a non-toxic agent is used so as to prevent adverse tissue reactions when the hydrogel is placed into the eye (Hoare and Kohane, 2008).

The stability of a chemically cross-linked hydrogel was demonstrated by Yu et al. (2015). In this study a hydrogel comprised of hyaluronic acid and dextran was evaluated for the delivery of bevacizumab, a monoclonal antibody which is used to treat neovascular diseases (Grisanti and Ziemssen, 2007). The hydrogel system was designed so that once it had been injected intravitreally, the polymers would form a solid gel. While this delivery system design has the benefits of a chemically cross-linked hydrogel, it also does not contain any cross-linking agent (the polymers cross-link with each other in response to physiological conditions) thereby improving its biocompatibility. The hydrogel system was able to release the active ingredient via a controlled release mechanism and maintain a therapeutically relevant concentration within the vitreous over a period of 6 months during *in vivo* studies. This would eliminate the current monthly schedule needed for bevacizumab administration, the risks of which have been discussed above (Yu et al., 2015).

## Stimuli-Responsive and *In Situ* Hydrogel Systems and Their Applications in Ocular Drug Delivery

*In situ* forming gel preparations offer an interesting advancement in sustained drug release profiles. This can be particularly useful in terms of the delivery of drugs to the eye as these systems provide an increased retention time at the cornea as well as prevent the rapid removal of the formulation via the nasolacrimal drainage system (Cheng et al., 2016). Both of these factors play a role in overcoming the current challenge of low bioavailability seen in many ocular drug delivery preparations.

**FIGURE 3:**
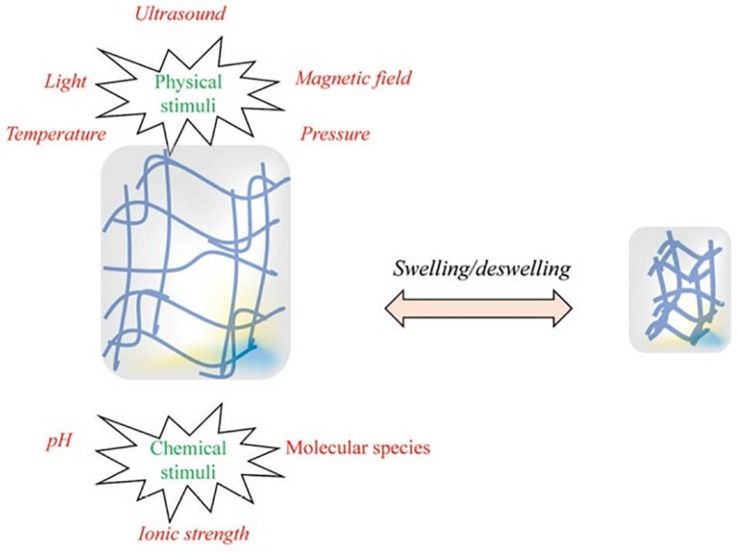
Illustration of the chemical and physical stimuli to which a hydrogel can respond. These stimuli are able to be provided by the body (for example, temperature and/or pH changes between conditions under which the hydrogels are stored and the conditions of the site into which it is administered) or externally (for example, ultrasound waves or a magnetic field). These stimuli can cause or a hydrogel to swell or de-swell, depending on how the formulation is designed. Reversible hydrogels are able to return to their original state when the stimulus is removed (adapted with permission from Fathi et al., 2015).

These *in situ* gelling systems are a type of stimuli-responsive hydrogels that are able to be administered to the eye as a liquid drop and subsequently form a gel after administration; known as a sol–gel transition. Gelation can be brought about as a response to a change in pH, ionic content, or temperature; although not all hydrogel systems are designed as stimuli-responsive systems and are simply administered as a gel (Al Khateb et al., 2016). Along with the ease of administration and prolonged retention time, *in situ* gelling systems have other advantages such as accurate dosing, simple formulation processes, and easy sterilization (Agrawal et al., 2010). Figure 3 depicts the various stimuli which can cause a hydrogel to swell or de-swell.

*In situ* gelling systems have also been shown to exhibit sustained drug release profiles, another beneficial factor in ophthalmic drug delivery. This has been observed in many of the studies which are discussed below.

### Temperature-Sensitive Hydrogel Systems

Temperature-sensitive, also known as thermosensitive, hydrogels undergo swelling or de-swelling in response to a change in temperature. There are three classifications of thermosensitive hydrogels; negatively thermosensitive (these contract in response to an increase in temperature), positively thermosensitive (these contract in response to a decrease in temperature), and thermally reversible gels (Masteikova et al., 2003).

Thermosensitive *in situ* hydrogels, which are commonly utilized for drug delivery purposes are liquid at room temperature (20–25°C) and form viscous gels at body temperature (34–37°C). The polymers used in these systems have a lower critical solvent temperature; the temperature at which the sol–gel transition occurs. It is important that this critical temperature is close to bodily temperatures so that the systems do not require an external heat source to form a gel (Kumar et al., 2013). The thermosensitive properties of these hydrogels have also be proven to be beneficial in recent cartilage tissue engineering research as they allow for minimally invasive administration yet form a scaffold with suitable mechanical strength. These systems are also able to mold into the irregular shaped area into which they are administered (Wang et al., 2019).

An *in situ* thermosensitive hydrogel was developed by Chen et al. (2012) for the delivery of a model drug, levocetirizine dihydrochloride. The hydrogel system was comprised of chitosan and disodium α-D-glucose-1-phosphate (DGP) and showed many favorable results. The formulation was a low viscosity liquid at room temperature and a gel at physiological temperature. It showed an initial rapid release of the drug, followed by a sustained drug profile. When in a gel form, the system showed that it had a prolonged residency time, in comparison to that of an aqueous solution, as well as improved cornea penetration of the drug (Chen et al., 2012). This shows that a thermosensitive hydrogel system is able to overcome some of the challenges seen in conventional ophthalmic treatments.

### pH-Sensitive Hydrogel Systems

These *in situ* gelling systems either swell or de-swell as a response to a change in the pH within the environment into which it is placed. The polymers used in pH-sensitive hydrogels have ionic groups which give them their responsive ability. For example, cellulose acetate phthalate latex (formulation pH of 4.4) has been shown to form a viscous gel when placed into the cul-de-sac of the eye. However, the development of pH-sensitive gels must take into account the delicate environment of the eye. The formulation must have a buffer capacity that can form a gel when placed into the eye but not cause damage to the eye (Kushwaha et al., 2012).

Although many of the polymers used in pH-sensitive hydrogels are synthetic polymers, such as carbopol [polyacrylic acid (PAA)] and polyethylene glycol, natural biopolymers are also used in the formulations to give them more favorable characteristics (Kushwaha et al., 2012; Wu et al., 2013). For example, in a study performed by Kumar and Himmelstein (1995), it was shown that, although PAA is able to change from a low viscosity liquid when in an acidic solution to a gel at a higher pH, the amount of PAA needed for this to occur was too high. This means that the solution could not be neutralized by the tear fluid which acts as a buffer in the eye. To overcome this, hydroxymethylcellulose, a natural polymer also able to act as a viscosity modifier was added. Both the PAA and the hydroxymethylcellulose were low viscosity liquids at pH 4.0 and transformed into viscous gels at a pH of 7.4. This meant that the concentration of PAA could be reduced to a safe level, without compromising the gelling and rheological behavior of the system (Kumar and Himmelstein, 1995).

The ability of methylcellulose, as hydroxypropylmethylcelullose, to act as a viscosity modifier in a pH-sensitive gelling system was further demonstrated by Srividya et al. (2001). The researchers developed a pH-triggered *in situ* gelling system comprised of PAA and hydroxypropylmethylcellulose which was shown to be a viable system in the topical delivery of ofloxacin.

### Ion-Sensitive Hydrogel Systems

An ion-sensitive gel transforms from a liquid to a gel as a result of a change in ion concentration within the environment it is exposed to. An example of such a gel is shown in a study by Liu et al. (2006). The researchers formulated an alginate hydrogel for the delivery of gatifloxacin, a broad-spectrum antibiotic, which underwent a sol–gel transition when exposed to divalent cations. Methylcellulose was incorporated in order to decrease the amount of alginate needed for gelation. This formulation was able to release the active ingredient over an 8-h period *in vitro* and formed a gel within the cul-de-sac of the eye when administered as a drop. This renders an ion-sensitive hydrogel a suitable alternative to conventional eye drops as it increased the residence time and sustained drug release profile will lead to an improved bioavailability (Liu et al., 2006).

### Ultrasound-Responsive Hydrogel Systems

Ultrasound responsive systems are able to deliver drugs to a specific site which prevents the side effects which can be seen with systemic administration of certain drugs. These systems can incorporate nanotechnology. Polymeric hydrogels or nanocarriers such as nanobubbles are loaded with the drug and, once administered, exposed to ultrasound waves. This then leads to cavitation and high temperatures at the site, causing the rupture of the polymeric chains of the nanobubble (Mura et al., 2013; Mahlumba et al., 2016).

Ultrasound-responsive systems are able to deliver a drug at a rate which is controlled from an external source which make them particularly useful in the investigation of cancer treatment. An example is the use of oxygen nanobubbles used for the delivery of mitomycin-C. The nanobubbles system was capable of lower tumor progression rates with a 50% lower drug concentration (Bhandari et al., 2018).

The application of ultrasound waves has been shown to be beneficial in the penetration of drugs through the various barriers of the eye, including the cornea. This was shown to be true in a study performed using dexamethasone where a significant increase in the permeability of the cornea was observed (Nabili et al., 2013). However, there is some concern over the increase in temperature which is induced as it may cause damage to the sensitive structures within the eye. A study was completed by Nabili et al. (2015), which showed that the ultrasound frequency which had previously been shown to increase penetration was safe for the ocular tissues tested.

### Iontophoresis: An External Stimulus for More Effective Ocular Drug Delivery

Iontophoresis is a physical force-based response technique which is used to enhance the penetration of an ocular active ingredient through the various tissue layers found in the eye. This is done by applying an electric current between two electrodes; one which is used to deliver the drug and another which is placed on the body. The ionized drug is then able to travel through the tissue as a conductor of the current. Iontophoresis has been illustrated extensively in transdermal applications but has also been investigated for use in ocular drug delivery (Eljarrat-Binstock and Domb, 2006).

There are many challenges, which have highlighted throughout this article, associated with the delivery of drugs to the anterior chamber of the eye but there are even more challenges in the delivery to the posterior segment. Most active ingredients aren’t able to penetrate through to the posterior segment when they are applied topically. This has led to the investigation of alternative routes of delivery such as intravitreal, subconjunctival, or transscleral. Iontophoresis has also been considered to aid in delivering drugs to the posterior segment. This allows for the treatment of conditions such as retinitis, uveitis, diabetic retinopathy, and age-related macular degeneration (Myles et al., 2005).

There are various device designs which can be utilized for iontophoresis; one such design includes a hydrogel. A hydrogel pad is saturated with a drug and acts as the delivery probe. This system has been shown to have promising results when tested with various drug entities such as dexamethasone. Transscleral hydrogel-based iontophoresis devices have been tested in both *in vivo* studies and clinical trials in healthy subjects and have shown good safety profiles as well as successful delivery of drug to the retina and choroid (Huang et al., 2018).

Although there are some iontophoresis devices which have been designed for transscleral drug delivery, the process does have some disadvantages. As with any medical procedure, there are risks involved; these include epithelial edema, inflammation, and burns (depending on the current density and duration of treatment). Iontophoresis has been demonstrated to be effective in improving the penetration of steroids, antibiotics, and antivirals. However, it has been reported that it is not able to deliver macromolecules to the vitreous in rabbits at a significant concentration (Thrimawithana et al., 2011).

In a study by Eljarrat-Binstock et al. (2008), hydrogel iontophoresis was employed to deliver nanoparticles to the eyes in an *in vivo* rabbit model. This study also investigated whether positively or negatively charged fluorescence nanoparticles penetrated through the tissues better. The researchers noted that, while iontophoresis is effective in improving the penetration of drugs into the eye, each active ingredient needs to be evaluated separately due to the fact that the physicochemical properties of the molecule will influence its behavior during the procedure. In this study, the, respectively, charged nanoparticles were loaded into a hydrogel sponge and were administered via an iontophoretic device at the central cornea and at the sclera. After a specified amount of time the eyes of the rabbits were enucleated and tissue samples collected. The negatively charged particles showed penetration into the inner ocular tissues after 4 h, which increased after 12 h. However, the positively charged nanoparticles showed extensive penetration into the inner tissues at just 4 h after administration, illustrating the effect of the physicochemical properties of the particles on their behavior. Both of these indicate that iontophoresis is an effective way of ensuring the penetration of nanoparticles (which are able to be loaded with an active ingredient) through the eye (Eljarrat-Binstock et al., 2008).

Iontophoresis has also been used for the delivery of drugs through the suprachoroidal space (SCS). In a study performed by Jung et al. (2018), a micro-needle device was tested for the delivery of nanoparticles in an *ex vivo* rabbit model. The results showed that with an injection into the SCS without iontophoresis the nanoparticles that were localized around the site of injection (less than 15% delivered to the posterior region of the SCS). However, in the eyes on which iontophoresis was performed, over 30% of the nanoparticles were found in the posterior region of the SCS; this was also found in the *in vivo* study. These studies show how iontophoresis is able to improve the delivery of drugs to the eye and is able to be used in place of other delivery systems such as intravitreal injections (Jung et al., 2018).

## Biopolymers Employed in the Formulation of Ocular Hydrogel Systems

Natural polymers have been widely investigated in a number of medical fields, including tissue engineering and drug delivery. This is largely due to the fact that they are biodegradable within the body and do not induce an inflammatory reaction (Singh, 2011). In terms of tissue engineering, they have also been shown to be conducive to cell growth and have a structure similar to the tissue matrix (Zhang et al., 2019). This section will focus on how natural polymers are employed in drug delivery systems.

**FIGURE 4:**
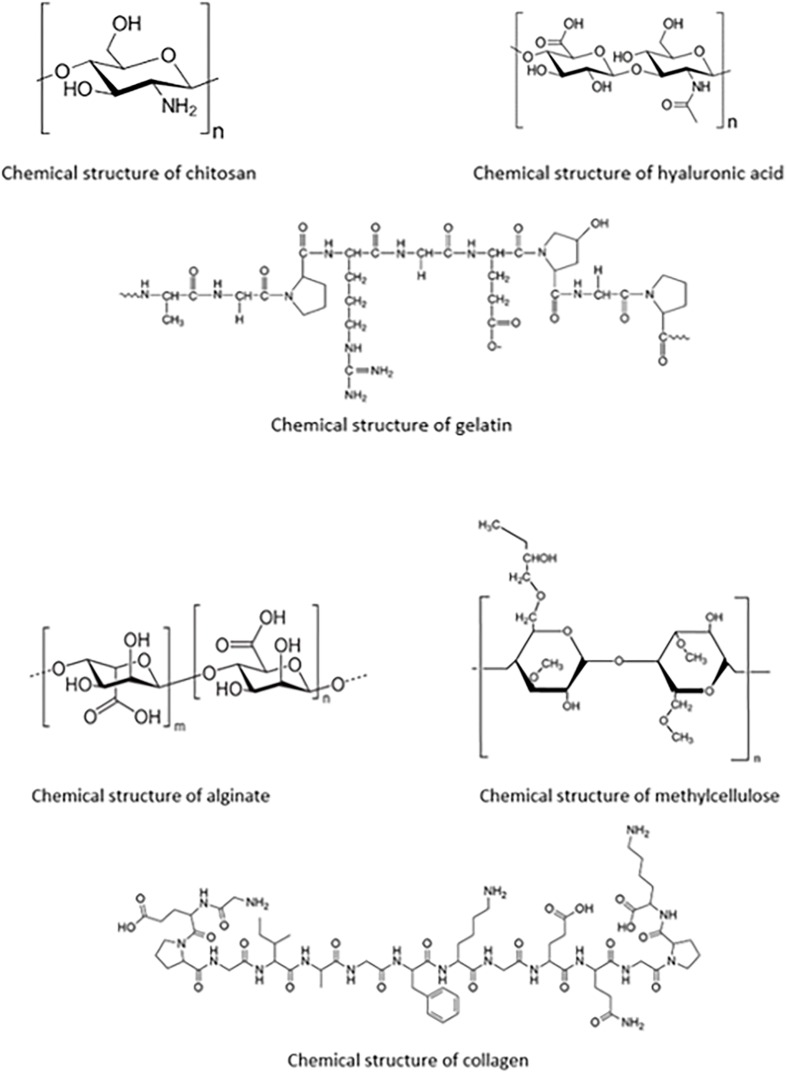
Chemical structures of each of the biopolymers; chitosan, hyaluronic acid, gelatin, alginate, methylcellulose, and collagen, for ocular polymeric drug delivery.

These polymers, also known as biopolymers, have long been viewed as a crucial aspect in the developments that are achieved in the field of drug delivery. Highlighted below are biopolymers commonly used in ocular drug delivery systems. Their chemical structures are shown in Figure 4.

### Chitosan Polymeric Bio-Platforms

Chitosan is one of the most widely used polymers in polymeric drug delivery systems due to its biocompatibility, biodegradability, and low toxicity profiles (Bhattarai et al., 2010). It is a cationic polysaccharide which is derived from chitin. One of chitosan’s most beneficial qualities is its mucoadhesive properties. The mucoadhesion is due to the fact that the positively charged chitosan is able to interact with the negative charges found in mucin (Fulgencio et al., 2012). This quality allows for improved permeation of drugs through ocular tissues as well as their controlled release from the formulation; both of which are vital in improving the delivery of drugs to the eye (Duttagupta et al., 2015).

Although chitosan is a very useful biopolymer for drug delivery, it is only soluble in acidic solutions. This is not desirable, especially when it is being formulated in ophthalmic formulations. For this reason, chitosan is often modified, for example through PEGylation and carboxymethylation (Xu et al., 2013).

A thermosensitive chitosan-based hydrogel was formulated by Cheng et al. (2016). This system was designed to overcome some of the challenges seen with latanoprost eye drops such as unwanted side effects after long-term use and low bioavailability. The hydrogel was characterized using both *in vitro* and *in vivo* tests for drug release and biocompatibility. The system was shown to be well tolerated and non-cytotoxic. During the *in vivo* studies, using a rabbit model, latanoprost was found in the aqueous humor 7 days after a single topical administration of the system, suggesting that this system could be administered on a weekly base instead of a daily basis as the commercial product is currently (Cheng et al., 2016).

Chitosan is often used in combination with other natural or synthetic polymers. For example, a study was performed by Cao et al. (2007) where a poly(*N*-isopropylacrylamide)-chitosan (PNIPAAm-CS) polymer was formulated into a thermosensitive *in situ* gelling system for the topical delivery of timolol, an active ingredient used for the treatment of glaucoma. The PNIPAAm-CS delivery system showed a higher Cmax and area under the curve (AUC) of blood concentration against time than that of a convention eye drop containing timolol. The gel system was also able to lower the intraocular pressure more than the eye drop over a 12-h period (Cao et al., 2007).

Another example is a hydrogel system was developed by Yu et al. (2017) containing carboxymethyl chitosan and a poloxamer composed of poly (ethylene oxide)/poly (propylene oxide)/poly (ethylene oxide) (PEO–PPO–PEO). The hydrogel was chemically crosslinked using glutaraldehyde and was able to undergo a reversible sol–gel transition in response to a change in pH and/or temperature. Preliminary studies, including cell studies performed with human cornea epithelial cells, showed that the hydrogel was not cytotoxic and has sustained drug release profiles (in comparison to a sample drug solution systems). This shows that this system could be further developed for ocular drug delivery (Yu et al., 2017).

### Hyaluronic Acid Polymeric Platforms

Hyaluronic acid is an anionic biopolymer which is found naturally within the human body. It is biodegradable and does not cause an immune response when used in medical systems. Due to this, hyaluronic acid has been a major interest in the design of drug delivery systems. It is particularly useful in respect to ocular drug delivery because it is a component within the vitreous humor of the eye and also has ligands for receptors found in many types of retinal cells, such as CD-44 (Martens et al., 2015).

Hyaluronic acid is endogenous to the body, making it highly biocompatible and non-immunogenic. However, it is not able to form a gel on its own and thus hydrogels made from hyaluronic acid rely on chemical modifications and cross-linking or gelling agents. Hyaluronic acid hydrogels have been investigated as a drug delivery system because they are able to be formulated as both static and stimuli-response (Trombino et al., 2019).

**FIGURE 5:**
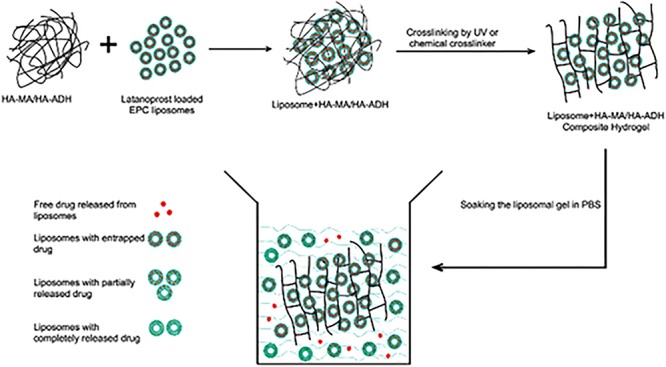
Drug release mechanism from hyaluronic acid-based nanocomposite hydrogel system. The active ingredient is loaded within the liposomes which are in turn loaded into the hydrogel. The drug is then released from the liposomes and diffuses through the hydrogel. It was also found that liposomes themselves were able to be released from the hydrogel. Both of these release mechanisms resulted in the sustained drug release seen in the formulation. This figure also highlights how the liposomes were incorporated into the hydrogel before it was cross-linked (adapted with permission from Widjaja et al., 2015).

Hydrogels are able to be utilized in conjunction with other technologies in order to improve ocular drug delivery. This can be seen in a study by Widjaja et al. (2015), where a hyaluronic acid-nanocomposite hydrogel was formulated with a sample drug, latanoprost. This system, in which the modified hyaluronic acid was combined with liposomes which contained the drug before crosslinking occurred, showed longer drug release profiles than the hydrogel and liposomes each did on their own. The composite system also improved the stability of the liposomes and the viscosity of the formulation. The hyaluronic acid was modified in two ways, using either adipic dihydrazide (ADH) or methacrylic anhydride (MA). Both modifications were tested throughout the study. The drug release mechanism is shown in Figure 5; it was found that both liposomes with entrapped drug and free drug were released from the hydrogel matrix which is what is believed to be the reason behind the sustained drug delivery profile which was observed. Although only preliminary studies were conducted; with further research, these nanocomposite systems are a potential candidate for the delivery of drugs to the eye after a single administration (Widjaja et al., 2015). Figure 5 shows how the drug is released from the system.

Another hyaluronic acid-based hydrogel system was developed by Wu et al. (2013). This system was designed to be a thermoresponsive microgel for the topical delivery of drugs to the eye. Hyaluronic acid was coupled with g-poly(*N*-isopropylacrylamide) to form HA-g-PNIPAAm which was shown to have high drug loading capabilities. The gel was tested for biocompatibility in rabbit eyes with the results showing that is was safe and did not cause any irritation. The formulated system, with a sample drug cyclosporine A (CyA), was tested against a castor oil solution of CyA and a commercial product also containing CyA. There was a significantly higher concentration of CyA in the corneas of rabbits who received the HA-g-PNIPAAm system than in those who received the other two solutions. This shows that *in situ* thermoresponsive gels are able to improve the bioavailability of ocular active ingredients (Wu et al., 2013).

Hyaluronic acid hydrogels have been investigated not only as a drug delivery system but also as an artificial vitreous substitute. Schramm et al. (2012) completed a study whereby hyaluronic acid hydrogels were formulated using two different cross-linking methods; the first through the use of dihydrides as a cross-linking agent and the second through photocrosslinking. Both methods resulted in three-dimensional hydrogels which had suitable optical transparency and rubber-like consistency. The results of this study showed that these hydrogels are able to replace the conventionally used silicone oils, which have disadvantages such as the formation of cataracts and a need for surgical removal of the oil, as a vitreous replacement on a long-term basis (Schramm et al., 2012).

### Gelatin Polymeric Platforms

Gelatin is a natural polymer which is biocompatible and biodegradable. It is derived from collagen, a substance which is found naturally within the stroma of the cornea and sclera. It has been investigated for a number of ocular drug delivery systems; including nanoparticles (Vandervoort and Ludwig, 2004). Natu et al. (2007) performed a study where gelatin hydrogels were investigated as a drug delivery system for pilocarpine, an ocular active used in the treatment of glaucoma. The hydrogels were formulated through chemical crosslinking with *N*-hydroxysuccinimide (NHS) and *N*, *N*-(3-dimethylaminopropyl)-*N*′-ethylcarbodiimide hydrochloride (EDC). These crosslinkers were used in a variety of concentrations which altered the degree of crosslinking and subsequently the release of the drug from the hydrogel. The release of pilocarpine from the various hydrogels ranged from 29.2 to 99.2% over an 8-h period. The hydrogels also displayed good adhesion and non-cytotoxicity profiles. This shows hydrogels comprised of gelatin to be a viable option for the delivery of drugs to the eye (Natu et al., 2007).

**FIGURE 6:**
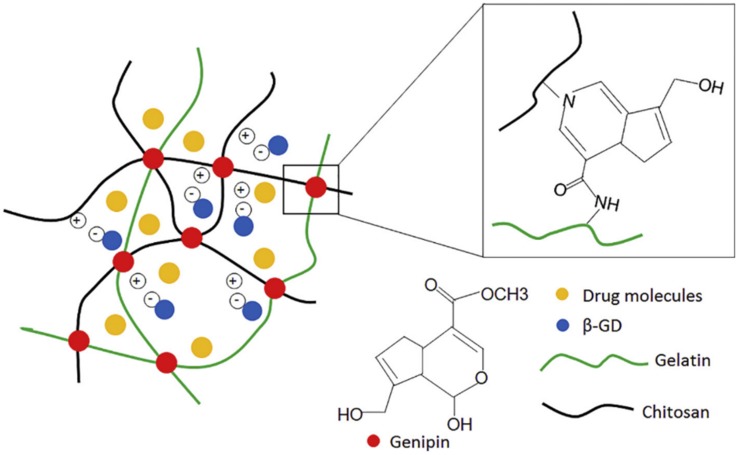
Illustration of the double crosslinking method using β-glycerophosphate disodium and genipin. The β-glycerophosphate disodium negatively charged phosphate groups underwent electrostatic attraction to the positively charged chitosan which gave this formulation the ability to transition between a solution and a gel (adapted with permission from Song et al., 2018).

In a study by Song et al. (2018), chitosan and gelatin were used to form a hydrogel aimed at improving the sustained delivery of drugs to the eye. The hydrogel was formed using a double crosslinking method; using both genipin and β-glycerophosphate disodium salt hydrate as crosslinking agents. The resulting hydrogel had *in situ* gelling properties; showing rapid gelation at 37°C. Timolol maleate was used as a sample drug as a comparison could be made against a commercially available product. The hydrogel delivery system was non-toxic and showed a sustained release drug release profile. During *in vivo* studies, in comparison to the commercial product, the hydrogel delivery system was able to show a longer lasting and more effective reduction (due to a twofold increase in duration) in the intraocular pressure. The *in situ* gelling property also prevented the system from being rapidly removed from the lower conjunctival sac by tears following administration (Song et al., 2018). Figure 6 shows the double crosslinking-method which is used in this formulation.

### Alginate Polymeric Platforms

Alginate is another highly biocompatible polysaccharide that is able to undergo ion-responsive gelation (Liu et al., 2008). It is classified as a polyanionic copolymer and is extracted from brown sea algae. Alginate forms a hydrogel when it is exposed to divalent cations such as Ca^2+^ (Lin et al., 2004). It has been used in ocular hydrogel preparations because it is non-cytotoxic and biodegradable. It was used in a formulation by Lin et al. (2004) which is discussed below under “ion-sensitive hydrogels.”

The utilization of alginate can also be seen in the study reported by Mandal et al. (2012) where an *in situ* forming gel was prepared using sodium alginate for the sustained delivery of moxifloxacin hydrochloride, a broad spectrum antibiotic. In this formulation, although sodium alginate was used as the primary gelling polymer, hydroxypropyl methylcellulose (HPMC) was also added as a viscosity enhancer. The resultant formulation was able to lengthen the precorneal residence time of the drug (also due to sodium alginate’s mucoadhesive properties) and improve its bioavailability. The polymer was able to undergo a sol–gel transition in response to an ion exchange when administered to the eye. *In vivo* studies were performed for biocompatibility using healthy male albino rabbits. The rabbits showed no signs of irritation after the formulation was administered to the eye and no ophthalmic damage was noted. This makes this formulation a viable alternative to conventional eye drops for the delivery of moxifloxacin with a less frequent dosage schedule (Mandal et al., 2012).

Sodium alginate hydrogels have also been used in the delivery of anti-inflammatory drugs to the eye. One such formulation is that prepared by Pandit et al. (2007). They highlighted the preference for hydrogel systems over implants as novel ocular drug delivery systems due to the fact that hydrogels are more cost effective and comfortable to the patient while still overcoming the bioavailability issues that are seen with convention drug delivery systems. The hydrogel which was produced supported these sentiments; sodium alginate was formulated into an *in situ* gelling system which would increase the residency time of the drug as well as exhibit sustained drug release profiles; both of which are vital in improving the bioavailability of ocular drugs (Pandit et al., 2007).

### Methylcellulose Polymeric Platforms

Methylcellulose is natural polymer which is often used as a viscosity enhancer in ocular formulations. It is capable of undergoing a reversible sol–gel transition when it is heated. This makes it useful in the development of *in situ* gelling hydrogel systems (Sultana et al., 2006a).

In a study by Silva et al. (2017), a HPMC hydrogel was used to aid in the delivery of chitosan-hyaluronic acid nanoparticles to the eye, giving another example in how a hydrogel can be employed in a drug delivery system. Methylcellulose was used because it is safe to sterilize within an autoclave, it has a suitable pH for the eye and has been shown to be used successfully in other ophthalmic preparations (Silva et al., 2017). This study highlights one of the derivatives of methylcellulose, among others, which are often used in preparations. This is due to the fact that these derivatives influence the temperature at which the methylcellulose is able to undergo a sol–gel transition. For example, by lowering the molar substitution of hydroxyl propyl groups, the transition temperature is reduced from between 75 and 90 to 40°C (Gambhire et al., 2013).

Methylcellulose can also be added to a formulation to adjust its gelation behavior. This was investigated by Dewan et al. (2015) in a study where methylcellulose of varying molecular weights were added to Poloxamer 407 (PM), a polymer previously investigated for the delivery of various drugs to the eye. However, when used in these formulations, PM is diluted by the lacrimal fluid of the eye and loses its ability to form a gel. Increasing the concentration of PM is not a viable solution as it causes the gelation temperature to drop; resulting in the formulation turning into a gel at room temperature. It was found that the addition of methylcellulose resulted in a decrease in the gelation temperature of the PM formulations and facilitated extended drug release profiles of the sample drug; making it a viable option for sustained drug delivery to the eye (Dewan et al., 2015).

A further study which illustrates that methylcellulose can be utilized in ophthalmic drug delivery preparations is that performed by Bain et al. (2009). Agents such as fructose and sodium citrate tribasic dehydrate were added to the formulation to reduce the gelation temperature. These additives have an impact on the gelation temperature by affecting the interactions between the polymer and the water molecules. The sample drug used was ketorolac tromethamine (KT). The resulting formulation was able to extend the release of the drug from 5 to 9 h, largely due to the presence of fructose which further enhances the viscosity of the formulation. Although further testing and *in vivo* studies are needed, the resulting formulation is a viable option for the delivery of drug to the eye in the place of conventional eye drops (Bain et al., 2009).

### Collagen Polymeric Platforms

Collagen is a natural polymer which is also available to be used in ocular drug delivery systems. Type 1 collagen is one of the primary components of the cornea and has been used in scaffolds for tissue engineering (Chen et al., 2005). Collagen shields have been formulated and are able to deliver drugs to the eye for a maximum of 72 h. This is more beneficial than soft contact lenses, which have been shown to only delivery the drug for the first 1–2 h after insertion. These shields are generally used following ophthalmic surgery for the delivery of anti-inflammatory or immunosuppressive active ingredients, among others. However, these shields are non-transparent and have to be applied by a surgeon (Liu et al., 2008).

However, there are some collagen shields available which have the potential to be self-administered. As reported by Khan and Khan (2013), these bandage contact lenses are able to facilitate the healing of the cornea following surgery or injury by protecting it from abrasion caused by blinking. They are also able to be laden with active ingredients; as the tears dissolve the contact lens, the drug is released along with a layer of collagen which is able to lubricate the eye. This provides a system which is able to increase the residency time of the drug at the cornea, allowing for increased permeability and bioavailability (Khan and Khan, 2013).

An example of a formulation where collagen, along with hydrogel technology, has been developed is that reported by Liu et al. (2006) where composite collagen hydrogels were formulated which contained alginate microspheres for the delivery of drugs to the eye. The composite hydrogels were characterized and shown to be suitable for use in ocular inserts or contact lens formulations as they were biocompatible and showed sustained drug release profiles as well as supported the attachment and growth of corneal epithelial cells (Liu et al., 2006).

Collagen has also been used in hydrogels that are intended for tissue engineering purposes. They have been investigated as an alternative to amniotic membrane which is used for clinical ocular surface reconstruction. This is due to the fact that they biodegrade at a suitable rate and offer very low immunogenicity. In a study by Mi et al. (2010), these collagen-based scaffolds were investigated. It was found that collagen gels are difficult to manipulate because of their weak structure. This was overcome through controlled unconfined plastic compression which, depending on the collage concentration and time for which the gel was compressed, produced a scaffold which closely mimiced the structure of the cornea. These hydrogel scaffolds were able to adequately support cell attachments and epithelial cell growth (Mi et al., 2010).

## Safety by Design of Polymeric Hydrogels Through Ocular Biocompatibility and Biodegradation

The eye is an organ of immune privilege, which protects its visual capability from the potentially sight-threatening sequelae of intraocular inflammation (Keino et al., 2018). Consequently, any potential formulations used in the eye, whether it be for drug delivery, tissue engineering, or any other medical procedure need to be vigorously tested for biocompatibility.

### Biocompatibility

Many studies in which new ophthalmic formulations are being investigated include biocompatibility studies. Typically, the first step in determining biocompatibility is to determine the cytocompatibility of the formulation. This is done through cytotoxicity or cell proliferation tests which are performed *in vitro*. The cell line most commonly used for these tests is human corneal epithelial cells (HCECs). These *in vitro* tests are useful in determining biocompatibility as they provide a controlled environment whereby researchers can observe the impact of the polymers used in their formulation on cell characteristics such as adhesion, proliferation, and viability. It has been noted that cell studies which are performed with multiple, different cell lines provide a more accurate representation of the cells found within tissues than studies where only a single cell line is used (Huhtala et al., 2007).

The second process in determining biocompatibility is through *in vivo* testing. This is usually performed using animal models. The New Zealand white rabbit model is most commonly used in ophthalmic bioavailability studies. This is because the eye of an adult rabbit is big enough to ensure the procedure is performed accurately (for example, rat eyes are sometimes used but are often too small for formulations designed for use in human eyes) and there is no pigment epithelium in the eye (Short, 2008).

Although the majority of the studies that are detailed in this review include biocompatibility studies in addition to other characterizations, either through *in vitro* or *in vivo* testing, there are those available which focus primarily on biocompatibility. One such study is that performed by Lai (2010). The authors investigated the effect of different cross-linkers [namely glutaraldehyde (GTA) and 1-ethyl-3-(3-dimethyl aminopropyl) carbodiimide (EDC)] on the ocular biocompatibility of gelatin hydrogels. Gelatin has been shown to have a rapid dissolution when it has not been cross-linked and is placed within an aqueous environment, which would limit its potential application in the delivery of drugs to the eye. The biocompatibility was tested using both cell culture techniques and *in vivo* animal testing. The cell line selected was primary rat iris pigment epithelial cells; these were cultured and observed for cell proliferation, viability, and presence of pro-inflammatory genes.

The results showed that the EDC cross-linked gels were better tolerated than the GTA hydrogels. This was then corroborated in the *in vivo* tests whereby the gelatin hydrogels were inserted into the anterior chamber of the eye of New Zealand white rabbits and observed for 12 weeks. The rabbits who were given the GTA cross-linked hydrogels showed a significant inflammation reaction whereas the EDC cross-linked hydrogels were well tolerated, concluding that EDC is more suitable as a cross-linking agent for the formulation of ophthalmic gelatin hydrogels. This study highlights that, although gelatin itself is biocompatible, the cross-linking agents which are used in the formulation of hydrogels have the ability to change the biocompatibility of a formulation (Lai, 2010).

The results mentioned in the study above were further corroborated in another study; also focusing on the biocompatibility of GTA and EDC cross-linked hydrogels, with the exception of using hyaluronic acid as the polymer. The results of the *in vivo* tests, performed using rabbits, showed that the EDC crosslinked hydrogel elicited no inflammatory response whereas the GTA cross-linked hydrogels produced a severe tissue response. This further highlights the importance of biocompatibility testing, not only for the polymer, but also for the other reactants used within a formulation (Lai et al., 2010).

Other *in vitro* methods for testing biocompatibility have been developed. An example of this is the development of a three-dimensional, curved epithelium model which is able to mimic the cornea. This model was designed and created by Postnikoff et al. (2014) in the hopes of removing the need for the use of animal testing in the development of some ophthalmic preparations. This particular model was shown to be multi-layered and responsive to cytotoxic compounds, as a cornea would which makes it a viable option in the biocompatibility assessment of contact lenses (Postnikoff et al., 2014).

### Biodegradability

Biodegradability is one of the aspects which makes the polymers discussed in this review beneficial for use in ocular drug delivery. This allows sustained drug release systems to be able to breakdown and be absorbed by the body, eradicating the necessity for surgical removal. The most common form of biodegradable system is that where a drug is embedded within a polymeric system and is released as the polymer degrades. The advantage of biodegradable over non-biodegradable ocular systems has been seen in implants developed for sustained drug release. Majority of ocular implants currently available on the market are non-biodegradable but research is being done into the development of biodegradable formulations (Lee et al., 2010).

The biodegradable nature of polymers, while advantageous, can sometimes hinder their ability to maintain their integrity for an extended time within the environment into which they are placed. For example, hyaluronic acid, which is broken down by hyaluronidase, does not have a sufficient residence time for long-term delivery. Hyaluronic acid is often modified to overcome this issue (du Toit et al., 2013).

## Incorporation of Hydrogels and Nanotechnology for Ocular Drug Delivery

Hydrogels can form a vital role in the development of nanotechnologies for the delivery of drugs to the eye. An example of this is the formulation of hydrogel nanoparticles. This drug delivery system combines the benefits of a hydrogel (hydrophilic and high-water content) with the minute size of a nanoparticle. These have been developed using both synthetic and natural polymers but, in this article, only those employing natural polymers are discussed (Hamidi et al., 2008).

Although hydrogels themselves offer many advantages to overcome these challenges, by combining hydrogels in colloidal drug delivery systems the effective delivery of drugs to the eye is further improved. Nanotechnology, such as nanoparticles and nanoliposomes, has been given a lot of focus in recent years for use in ocular drug delivery. These nanocarriers are able to offer advantages such as the more targeted delivery of drugs and controlled release as well as reduced toxicity and improved efficacy of formulations. These carriers, which range from 1 to 1000 nm in size, are also able to deliver drugs which are poorly water soluble (a problem that in the past has seen ocular active drugs not being made into effective preparations) as well as provide improved penetration into tissues. Colloidal drug delivery systems are also able to increase the retention time at the surface of the cornea, resulting in improved bioavailability (Ameeduzzafar et al., 2016).

In terms of ocular drug delivery, nanoparticles are useful due to their small size which allows for targeted drug delivery and improved bioavailability. The drugs in these delivery systems can be incorporated into the nanoparticle either through entrapment, encapsulation, or attachment to the surface. Nanoparticles with intrinsic hydrogel structure are able to be formulated using either physical or chemical cross-linking methods and have been prepared using a number of synthetic and natural polymers. Nanoparticles are able to be combined with hydrogel technology either in the way that they are synthesized or in the way that they are administered where the hydrogel acts as a suspending agent (Hamidi et al., 2008).

A further example of the combination of hydrogels and nanotechnology is nanogels. These nanoparticle carriers have many beneficial properties in terms of ocular drug delivery. These include sustained drug delivery profiles and improved stability of the drug in water (Jamard et al., 2016).

In a study by Jamard et al. (2016), it was noted that many nanogels require harsh conditions for formulation, such as high temperatures and the use of organic solvents. However, it was noted that by using biopolymers (such as methylcellulose) which have been modified with hydrophobic moieties [such as poly(*N*-tert-butylacrylamide)], self-assembling nanogels could be formulated through hydrophobic interaction within an aqueous environment. This renders the resultant, non-cytotoxic nanogel suitable for the delivery of biological compounds with a prolonged release profile (Jamard et al., 2016).

A further study, focusing on the delivery of fluconazole to the cornea, was performed by Nishil et al. (2013) where fluconazole loaded chitin nanogels were synthesized. The system was shown to have sustained drug release drug profiles while also being cytocompatible. It was also noted that the system allowed for penetration through the cornea in *ex vivo* studies. The nanogel can be considered for improved bioavailability for the fluconazole in the treatment of corneal fungal infections (Nishil et al., 2013).

Solid lipid nanocarriers (SLN) are another form of nanotechnology which have been researched for the replacement of conventional ocular drug delivery systems. These SLNs are advantageous as they have low toxicity due to the fact that they are prepared from lipids natural to the body, are able to undergo autoclave sterilization, and are able to be loaded with both hydrophilic and hydrophobic drugs (Farid et al., 2017). SLNs fall under a larger group of lipid-based nanocarriers which also includes lipid-drug conjugates (Puglia et al., 2015).

Nanoparticles offer a particular benefit in that, due to the large surface area-to-volume ratio, they are able to support a vast number of surface functional groups (Jacob et al., 2018). These surface modifications are able to improve some of the disadvantages which are seen in certain nanotechnologies. An example of this can be seen in a study by Attama et al. (2008) where a phospholipid was used as a surface modifier on SLNs. The results showed that the drug release from the SLNs which were formulated without the phospholipid happened in a burst release fashion due to the fact that there was more drug present in the periphery of the nanoparticles. In addition, a large amount of drug was found in the bulk aqueous medium. Those that were formulated with the phospholipid had a sustained drug release profile. This illustrates how surface modifications are able to have an effect on not only the drug release profiles but also the encapsulation efficacy of SLNs (Attama et al., 2008).

The concept of colloidal nanoparticulate-based systems has been investigated for therapeutic contact lenses. The incorporation of nanoparticles allows for improved drug release from the contact lens as well as prevents the interaction of the drug with the polymers of which the lens is composed. An example of such system was formulated by Jung et al. (2013). Nanoparticles which contained timolol, a drug used to treat glaucoma, were loaded onto commercial contact lenses. The contact lenses were tested in preliminary drug release and *in vivo* studies which showed that, in addition of being biocompatible, they were able to release timolol over an extended period (5 days) resulting in a lowering of the intraocular pressure. These are promising results as an alternative to conventional timolol eye drops which must be administered multiple times a day; however, there is still further research which needs to be conducted (Jung et al., 2013). This research would include the impact of colloidal systems on the contact lens’ transparency and ion and oxygen permeability (Maulvi et al., 2016).

## Future Perspectives

The primary focus of the research that is being done, and that has been commented on in this article is to improve the shortfalls seen in current ophthalmic treatments. Whether that be the low bioavailability and rapid clearance from the administration site found with eye drop formulations or the frequency of invasive procedures seen with intravitreal injections, future developments made in ocular drug delivery are vital (Sapino et al., 2019).

Many of the advancements being made in this area of drug delivery include harnessing the benefits highlighted for both biopolymers and hydrogel systems. One of the main focuses of the future perspectives is the further testing of the systems that have been discussed in this paper. This testing includes *in vivo* animal testing of systems that have undergone cell testing, and clinical trials for the systems that have undergone animal pilot studies. It has been noted that not many of the newly developed systems have been made commercially available and these studies would further this process (Barbu et al., 2006).

Natural, biodegradable polymers have uses in other future prospects for ocular drug delivery outside of their use in hydrogel systems, both on their own and in conjunction with synthetic polymers. These include the development of polymeric ocular inserts [as an example, an insert was developed by Jain et al. (2010) with sodium carboxymethylcellulose and polyvinyl alcohol for the topical delivery of ciprofloxacin]. Majority of the ocular inserts which are commercially available are composed of synthetic polymers so the development and commercialization of biopolymer-based inserts is a definite avenue for the future prospects of biopolymer technology.

Hydrogel systems have been demonstrated in many studies to be highly beneficial in their role as ophthalmic drug delivery systems. The advances that have been made in recent years, particularly in terms of “smart” or stimuli-responsive hydrogels, have made a large impact. However, many of these formulations have not been made commercially available, mainly because many of them have yet to undergo clinical trials. This would be a vital step in improving the quality of life of patients; especially those who require eye drop administration on a daily basis. According to the research that has been done, hydrogels provide an option for far less frequent dosing schedules (in some cases weeks or months) (Chang et al., 2019).

## Conclusion

Although hydrogels are not as extensively investigated as some of the other developments that are being made in ocular drug delivery, they are making an impact. These systems provide two vital benefits to drug delivery; sustained drug release and increased retention time. They are able to be formulated in such a way that they are able to respond to stimuli, which has been shown to be very beneficial. This stimuli-response ability allows for ease of administration, making these formulations more favorable for patients. This takes the ease of administration of eye drops and combines it with the increased viscosity of ointments, resulting in effective topical drug delivery without frequent dosing schedules (seen with eye drops) and blurred vision (seen with ointments).

Biopolymers are at the forefront of many studies undertaken in ocular drug delivery. These polymers, with their non-cytotoxic, biodegradable profiles enable researchers to develop technologies without the risk of causing inflammation and the need for surgical removal. They also lend themselves to safety-by-design aspects for new formulations as there are many studies which illustrate their low toxicity profiles. Biopolymers provide an easily available and relatively cheaper option to some synthetic polymers.

Both hydrogels and biopolymers lend themselves to use in nanotechnology for ocular drug delivery. Whether it be in the form of the intrinsic make-up of the nanoparticles, nanoliposomes, or nanowires, or as a suspending agent, hydrogels can greatly impact the developments which are being made in this field of drug delivery. Although there are still developments to be made, both hydrogel and biopolymer technology play a vital role in the improvements being investigated for the effective delivery of drugs to the eye.

## Author Contributions

All authors listed have made a substantial, direct and intellectual contribution to the work, and approved it for publication.

## Conflict of Interest

The authors declare that the research was conducted in the absence of any commercial or financial relationships that could be construed as a potential conflict of interest.
